# Sexuality and Aging: Knowledge and Attitudes of Healthcare Practitioners in Colombia

**DOI:** 10.1093/geroni/igaf122.2774

**Published:** 2025-12-31

**Authors:** Maria Reyes, Natalie Levy

**Affiliations:** Universidad de los Andes, Bogota, Distrito Capital de Bogota, Colombia; Universidad de los Andes, Bogota, Distrito Capital de Bogota, Colombia

## Abstract

In Colombia, older adults face limited access to comprehensive care that included sexual health. Despite the importance of sexuality in older adults’ quality of life, healthcare professionals often overlook it in their assessments, reinforcing barriers in care. This study aims to identify the level of knowledge and attitudes of healthcare practitioners towards sexuality and aging. A cross-sectional quantitative study was conducted. The sample consisted of 150 healthcare practitioners in Colombia. The questionnaire included demographic data, education and experience, and the Aging Sexual Knowledge and Attitudes Scale, and the Staff Attitudes about Intimacy and Dementia scale.

**Results:**

professionals reported that they occasionally or rarely address sexuality-related topics with older adults in their practice. Additionally, participants indicated that they had not received training on these topics but expressed interest in doing so. The results showed a moderately positive trend in attitudes toward sexuality in later life. The highest agreement levels were found in the importance of sexuality for quality of life, the need to address it in healthcare, and its recognition as a relevant dimension of well-being. Participants reported a lack of confidence in addressing the topic in professional practice and perceived cultural barriers to discussing it, particularly concerning older adults with dementia and those in institutional settings. Discussion The findings suggest that healthcare professionals generally have positive attitudes toward sexuality in old age but struggle to address it in their practice. This difficulty may be related to the lack of specific training on the topic, which limits their confidence and knowledge.

